# Inflammation as a Therapeutic Target in Atherosclerosis

**DOI:** 10.3390/jcm8081109

**Published:** 2019-07-26

**Authors:** Mau T Nguyen, Sanuja Fernando, Nisha Schwarz, Joanne TM Tan, Christina A Bursill, Peter J Psaltis

**Affiliations:** 1 Vascular Research Centre, Lifelong Health Theme, South Australian Health and Medical Research Institute (SAHMRI), Adelaide, South Australia 5000, Australia; 2 Adelaide Medical School, University of Adelaide, Adelaide, South Australia 5000, Australia

**Keywords:** inflammation, atherosclerosis, C-reactive protein, canakinumab, methotrexate, colchicine, interleukin

## Abstract

Atherosclerotic coronary artery disease (CAD) results from build-up of cholesterol-rich plaques in the walls of the coronary arteries and is a leading cause of death. Inflammation is central to atherosclerosis. Uncontrolled inflammation makes coronary plaques “unstable” and vulnerable to rupture or erosion, leading to thrombosis and myocardial infarction (MI). As multiple inflamed plaques often co-exist in the coronary system, patients are at risk of repeated atherothrombotic cardiovascular events after MI, with rates of 10–12% at one year and 18–20% at three years. This is largely because current therapies for CAD, such as lipid-lowering statins, do not adequately control plaque inflammation. New anti-atherosclerotic agents are therefore needed, especially those that better target inflammation. The recent positive results for the anti-interleukin-1-beta (IL-1β) monoclonal antibody, Canakinumab, in the Canakinumab Anti-inflammatory Thrombosis Outcome Study (CANTOS) clinical trial has provided a major stimulant to the field. It highlights that not only is inflammation important from a pathogenic and risk prediction perspective in CAD, but that reducing inflammation can be beneficial. The challenge is now to find the best strategies to achieve this in real-world practice. This review outlines the role that inflammation plays in atherosclerosis and provides an update on anti-inflammatory therapies currently being investigated to target atherosclerosis.

## 1. Introduction

Cardiovascular disease (CVD) remains a leading cause of death, morbidity and health economic burden worldwide. It currently accounts for more than 17.6 million deaths each year and this number is projected to exceed 23.6 million by 2030 [1]. Atherosclerotic coronary artery disease (CAD), the underlying pathology in the majority of cardiovascular complications such as myocardial infarction (MI), is still the major cause of cardiovascular mortality [1]. One of the greatest triumphs of preventative cardiology has been the identification and subsequent aggressive control of traditional risk factors for atherosclerosis, such as hypertension, diabetes, cigarette smoking, and elevated low-density lipoprotein cholesterol (LDL-C). As a result, we have seen a dramatic decrease in the incidence of MI by up to 40% in many Western nations that have embraced a preventative approach to CAD [2]. One important example of this is the use of cholesterol-lowering hydroxymethylglutaryl-coenzyme A (HMG-CoA) reductase inhibitors, collectively known as statins, that reduce the incidence of major adverse cardiovascular events (MACE) by 25% to 40% in susceptible individuals with elevated LDL-C [3]. However, despite our best attempts to manage traditional risk factors with contemporary approaches, MACE continue to occur at unacceptable rates in patients with established CAD, with event rates of 10–12% at one year and 18–20% at three years after an index myocardial infarction (MI) [4]. This is largely because atherosclerosis is a complex disease process whose pathogenic basis extends far beyond intimal infiltration of cholesterol. In particular, numerous converging lines of clinical and experimental evidence show that inflammation is a key player in its initiation, progression and eventual thrombotic manifestations [5]. This review focuses on the role that inflammation plays in atherosclerosis and provides a timely update on the status of anti-inflammatory therapeutic approaches that are being investigated to target atherosclerosis and its lethal sequelae. 

## 2. Inflammation in the Pathophysiology of Atherosclerosis

Atherosclerosis is widely recognised as being a chronic inflammatory disease of blood vessels triggered by LDL-C accumulation, and characterised by formation of complex subintimal plaques that restrict luminal blood flow and have propensity to rupture or erode leading to thrombotic occlusion and tissue infarction. An early mechanism in atherogenesis is exposure of the intimal endothelium to noxious stimuli, such as hypercholesterolaemia, hypertension and pro-inflammatory cytokines, that disrupt its ability to function as a permeability barrier and cause it to become ‘activated’. In their activated state, blood vessels are prone to the uptake of lipoproteins, particularly ApoB containing LDL-C, from the circulating blood. These lipoproteins are subsequently retained in the arterial wall following interactions with subendothelial proteoglycans in the extracellular matrix, where they undergo chemical modifications. One such modification is oxidation which occurs secondary to interactions between lipoproteins and locally released reactive oxygen species (ROS), pro-oxidative enzymes and lipid oxygenases. 

**Figure 1:**
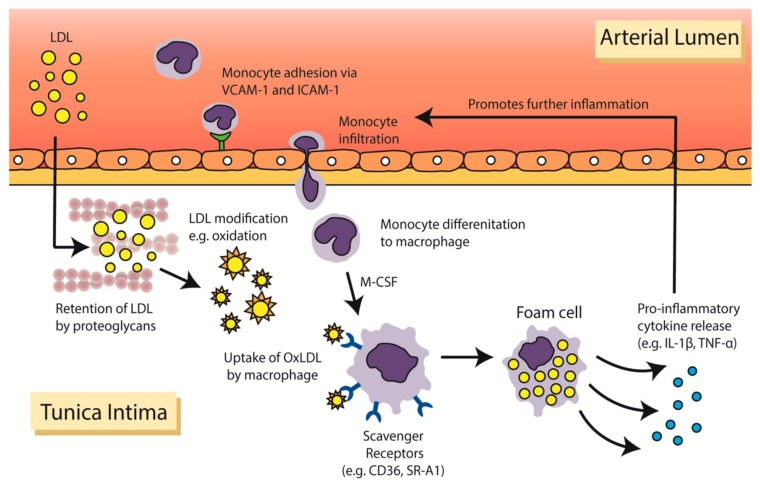
**Infiltration of low-density lipoprotein (LDL) and formation of macrophage foam cells in the arterial wall.** In individuals with hypercholesterolaemia, elevated levels of LDL-C are prone to infiltration and retention in the arterial wall. Monocytes recruited into the arterial wall differentiate into macrophages on stimulation by macrophage colony stimulating factor (M-CSF). Modified LDL particles are then taken up by macrophages via scavenger receptors. Accumulation of lipids in the macrophage results in the formation of lipid-laden foam cells leading to the release of pro-inflammatory cytokines. CD36, cluster of differentiation-36; ICAM, intercellular cell adhesion molecule 1; IL-1β, interleukin-1-beta; LDL-C, low-density lipoprotein cholesterol; M-CSF, macrophage colony stimulating factor; oxLDL, oxidised low density lipoproteins; TNF-α, tumor necrosis factor alpha; SR-A1, scavenger receptor type 1; VCAM-1, vascular cell adhesion molecule 1.

Another important feature observed consistently in early plaque formation is leukocyte recruitment to the vessel wall, which occurs in conjunction with lipoprotein uptake (Figure 1). After their activation, intimal endothelial cells, which normally resist the attachment of leukocytes in the bloodstream, increase their expression of leukocyte adhesion molecules, such as vascular cell adhesion molecule-1 (VCAM-1) and intercellular adhesion molecule-1 (ICAM-1) [6,7]. This resultant change in receptor expression promotes the extravasation of leukocytes, particularly neutrophils and monocytes, from the circulating blood into the subendothelial space. The initial adhesion of leukocytes to the activated endothelium is mediated by selectin-dependent adhesion molecules, such as E-selectin and P-selectin. Following this initial tethering, leukocytes form tighter adhesions via the binding of VLA-4 (very late antigen-4) on the leukocyte to its complementary ligands on the activated endothelial cell, VCAM-1 and ICAM-1. The importance of leukocyte adhesion in early plaque formation is supported by animal work showing significant reductions in lesion size in ICAM-1 and P-selectin null mice, as well as mice with genetic deficiencies in VCAM-1 expression [8,9,10,11].

Once resident in the arterial wall, monocytes differentiate into macrophages in the presence of growth factors, such as macrophage-colony stimulating factor (M-CSF) (Figure 1). Macrophages ingest modified LDL-C particles, including oxidised LDL (oxLDL), to become lipid-laden foam cells. The aggregation of foam cells results in a yellow coloured ‘fatty streak’ in the vessel wall and with it, the first macroscopic signs of atherosclerotic disease. Fatty streaks, also known as intimal xanthomas, have been identified in a third of children under the age of nine, and become increasingly prevalent by the age of 20–29 while remaining asymptomatic [12,13]. Uptake of oxLDL by monocyte-derived macrophages occurs primarily through a family of ‘scavenger receptors’ (SRs) that is upregulated during differentiation. Binding of oxLDL to SRs results in internalisation of these biochemically modified lipoproteins into cytoplasmic lipid droplets. While numerous SRs have been discovered, studies have shown that CD36 (cluster of differentiation-36) and SR-A1 (scavenger receptor type 1) account for up to 90% of oxLDL uptake by macrophages [14]. 

At first the initial uptake of oxLDL by macrophages to become foam cells is beneficial as it sequesters potentially damaging lipoproteins. However, over time the ability for foam cells to handle and process the lipoproteins is exceeded. This causes endoplasmic reticulum stress leading to ROS production which triggers an apoptotic cascade. While in part due to uncontrolled lipoprotein uptake, impairment of cholesterol efflux by foam cells also contributes to their demise [15]. This leads to the release of an array of pro-inflammatory cytokines, such as interleukin-1-alpha (IL-1α), IL-1β, IL-6, tumor necrosis factor-alpha (TNF-α) and matrix metalloproteinases (MMPs), which further exacerbates atherosclerosis by promoting immune cell infiltration. Furthermore, inefficient clearance of dead cell components, by a process called efferocytosis, can result in the accumulation of cellular debris and lipids. This predisposes the plaque to form a necrotic lipid-rich core that is highly thrombogenic [16].

The natural progression of atherosclerotic plaque from an intimal fatty streak to a fibrous atheromatous plaque is due to the recruitment of smooth muscle cells (SMC) from the tunica media—the middle layer of the artery wall–into the tunica intima. The migration of SMC occurs following the release of pro-inflammatory cytokines and proteolytic enzymes by activated endothelial cells, macrophages and foam cells that degrade the supporting extracellular framework. These cytokines then provide a chemotactic signal for the migration of SMC and stimulate a shift in their phenotypic profile from quiescent to actively synthetic. Once in the intima, SMC proliferate and produce an array of extracellular matrix (ECM) proteins, including type 1 and type 3 collagen, and proteoglycans that are thought to be pivotal to plaque remodeling and stabilisation [17,18]. The accumulation of SMC-derived ECM results in the formation of a fibrous cap that overlies the plaque. Collagen degradation leading to thinning of the fibrous cap has been found to be an important contributor to plaque rupture. 

While much has been elaborated on the role of macrophages in atherosclerosis, cells of the adaptive immune system, particularly T lymphocytes and B lymphocytes, are also important mediators of atherosclerosis [19]. Albeit at fewer numbers, these lymphocytes, particularly T lymphocytes, are seen in plaques as early as monocytes and appear to have key roles in regulating the inflammatory response elicited by macrophages during atherogenesis. T lymphocytes exhibit diverse functions in atherosclerosis. T-helper-1 (Th-1) lymphocytes secrete pro-inflammatory cytokines, such as interferon-gamma (IFN-γ), IL-2 and TNF-α, which activate macrophages, endothelial cells and SMC, therefore propagating and accelerating atherosclerosis [20]. On the other hand, regulatory-T (T-reg) lymphocytes are thought to downregulate the inflammatory process by secreting anti-inflammatory cytokines, such as IL-10 and transforming growth factor-beta (TGF-β) [21]. Similar to T lymphocytes, B lymphocyte functionality appears to be heavily dependent on the specific cell subtype. B1 lymphocytes, which may produce antibodies in response to an atherogenic antigen, appear to be protective against atherosclerosis [22,23], whereas B2 lymphocytes may exacerbate atherogenesis through the secretion of pro-inflammatory cytokines and activation of Th-1 lymphocytes [24,25]. The importance of the adaptive immune system has been demonstrated in experimental models of atherosclerosis which showed a 40% to 80% reduction in atherosclerotic disease burden in T and B lymphocyte deficient mice [26]. While further research is required to determine the extent of their involvement in atherosclerosis, it is likely that the net effect of T and B lymphocytes is dependent on the balance between their pro-inflammatory and anti-inflammatory subsets. 

## 3. Burden of Cardiovascular Disease in Chronic Inflammatory Diseases 

It has long been acknowledged that diseases characterised by chronic inflammation are disproportionately burdened by CVD with rheumatological conditions exemplifying this link [27]. Rheumatoid arthritis (RA) is a systemic autoimmune disorder characterised by inflammatory polyarthritis and progressive joint destruction that affects up to 1% of the general population [28]. Accumulating evidence indicates that individuals with RA have a significantly increased risk of mortality with CVD accounting for over a third of deaths [29,30]. Recent meta-analyses of observational studies report that RA increases the risk of incident cardiovascular events and cardiovascular-related mortality by 48% and 50% respectively, compared to the general population [31,32]. This is comparable to the magnitude of risk imparted on CVD by diabetes mellitus [33]. Specific to CAD, patients with RA are 68% more likely to have a MI compared to those without RA [31]. This result has recently been supported by a prospective population-based cohort study that reported a 43% increase risk of MI in patients with RA [34]. Furthermore, it appears that the increased incidence of CVD in patients with RA is independent of traditional risk factors [35]. This suggests that additional mechanisms account for the excess rates of CVD observed in RA with recent evidence implicating chronic inflammation as the most likely culprit [36,37].

In addition to RA, other systemic diseases characterised by chronic inflammation have also been associated with an increased risk of CVD [38,39,40]. These include psoriasis, ankylosing spondylitis, systemic lupus erythematosus as well as inflammatory bowel disease. The pathogenesis of these conditions share common inflammatory pathways to those involved in the development and progression of atherosclerosis. For example, the pathogenesis of psoriasis, which is an immune-mediated disease affecting the skin and joints, is primarily mediated by T lymphocytes; specifically, those displaying the Th-1 phenotype. Similar to Th-1 lymphocytes in atherosclerotic plaque, Th-1 lymphocytes in psoriatic plaques release pro-inflammatory cytokines, such as TNF-α, IL-1β and IL-6, that activate cells of the innate immune system such as macrophages and neutrophils [41]. Therefore, it is these shared pathways that may underlie and explain the increased rates of CVD observed in chronic inflammatory conditions. Furthermore, the discovery of auto-antibodies against oxLDL and growing evidence of autoimmune-like involvement of the adaptive immune system has led to consideration that atherosclerosis may in-part be an autoimmune condition or at least an auto-inflammatory mediated disease [42].

## 4. Beyond Statins: Where to Now for Anti-Atherosclerotic Therapies? 

As highlighted earlier, risk modification with statin therapy is now a common practice in both the primary and secondary prevention of atherosclerotic CVD. Statins have previously been shown to exhibit anti-inflammatory effects in addition to lowering lipid levels. Trials attempting to quantify overall inflammatory burden often utilise high sensitivity C-reactive protein (hsCRP), as it is an increasingly available and cost-effective test that captures most of the upstream inflammatory activity. The anti-inflammatory effect of statins was formally tested in the JUPITER (Justification for the Use of Statins in Prevention: An Intervention Trial Evaluating Rosuvastatin) trial [43]. In this study, participants with LDL-C levels < 3.4 mmol/L and elevated hsCRP > 2 mg/L were randomised to receive either 20 mg/day of rosuvastatin or placebo. Of the 17,803 apparently healthy individuals recruited, those treated with rosuvastatin had a 44% risk reduction in first ever cardiovascular events compared to the placebo group (HR 0.56; 95% CI 0.46–0.69; *p* < 0.00001). Relevant to the inflammation hypothesis of atherosclerosis, rosuvastatin was shown to lower hsCRP levels by 47% from a median of 4.2 mg/L at baseline to 2.2 mg/L at 12-month follow up. Therefore, it appears that patients who receive standard therapy with statins as part of secondary prevention may obtain some degree of anti-inflammatory coverage. Despite this, atherosclerotic events continue to occur at alarming rates, and it is thought that residual inflammatory risk may be an important driving force behind this. Analysis of the PROVE-IT (Pravastatin or Atorvastatin Evaluation and Infection Therapy) trial showed that of the 2099 patients who had a history of preceding acute coronary syndrome and were given 80 mg atorvastatin, 44% achieved reductions to target in both LDL-C (<1.8 mmol/L) and hsCRP (<2 mg/L) [44,45]. However, 29%, 13% and 14% of patients were left with suboptimal residual inflammatory risk (hsCRP > 2 mg/L), residual cholesterol risk (LDL-C > 1.8 mmol/L) or both, respectively. These results suggest that recurrent coronary events may be driven by different biological processes that require different management strategies to address. Using the same thresholds for residual risk as PROVE-IT, similar relative proportions were identified in secondary analyses of the IMPROVE-IT (Improved Reduction of Outcomes: Vytorin Efficacy International) trial [46]. This study randomised 15,179 stable patients with acute coronary syndrome to either 40 mg simvastatin or a combination of 40 mg simvastatin and 10 mg ezetimibe [47]; ezetimibe is a second line lipid-lowering drug that reduces intestinal absorption of cholesterol and is used as add-on therapy when response to statins is suboptimal or in the event of statin intolerance. The consistency of results from these studies suggest that despite statin and other lipid lowering therapies, residual inflammatory risk is common and over twice as prevalent as residual cholesterol risk. This is not surprising given that statin therapy has only relatively modest anti-inflammatory effects despite being highly effective in reducing LDL-C levels. Furthermore, results from IMPROVE-IT show that patients who achieved both a reduction in residual inflammatory risk and residual cholesterol risk had lower rates of the primary endpoint, defined as a composite of major coronary event, non-fatal stroke and cardiovascular death, compared to those who achieved reductions in only one residual risk [46]. Therefore, there is a clear need for future novel therapies to address the currently unmet issue of residual inflammatory risk in patients found to have persistent inflammation following standard care. 

## 5. Anti-Cytokine Therapy in Atherosclerosis: The Basis for Human Trials

The role of cytokines in atherosclerosis is complex with new discoveries constantly adding to our understanding of their function [48]. Cytokines are low-molecular weight proteins produced by various cell types that serve to mediate host inflammatory responses between cells [49,50]. Since the first ever description of interleukins in the early 1940s, cytokine classification has expanded to include over 30 interleukins, as well as branching to include other functional families of cytokines, including interferons, transforming growth factors and tumor necrosis factors. 

Human data implicating cytokines in the development and progression of atherosclerosis date back to cohort studies conducted two decades ago, of which the National Institutes of Health-funded Physicians Health Study [51] and Women’s Health Study [52] are most prominent. These studies found that elevated levels of circulating cytokines, such as IL-6, as well as elevated adhesion molecules, such as VCAM-1 and ICAM-1, were predictive of future myocardial infarction and stroke in apparently healthy individuals [51,52,53,54]. These results have subsequently been validated, with a recent meta-analysis concluding that other pro-inflammatory cytokines, including IL-18, MPP-9 (metalloproteinase-9) and TNF-α (tumor necrosis factor-alpha) are also associated with increased risk of MACE [55]. The IL-6 signaling pathway has emerged as an especially promising candidate for cytokine-based therapy, with Mendelian randomisation studies implicating it in both atherogenesis and acute plaque rupture [56,57,58]. IL-6 is produced by several cell types including monocytes, fibroblasts and endothelial cells upon stimulation by IL-1 [59]. IL-1β, the major circulating form of IL-1, is in turn activated by the NLRP3 (NOD-like receptor family pyrin domain containing 3) inflammasome. Inflammasomes are intracellular protein multimer complexes that play critical roles in the production of pro-inflammatory cytokines. The assembled NLRP3 inflammasome is formed from the combination of NLRP3, apoptosis-associated speck-like protein (ASC) and caspase-1. This complex activates procaspase-1 to caspase-1, which in turn converts pro-IL-1β and pro-IL-18 into their bioactive, pro-atherogenic forms, IL-1β and IL-18. Data linking IL-1β to atherosclerosis stem from early work demonstrated that a lack of IL-1β decreases the severity of atherosclerosis [60] and that administration of a monoclonal antibody targeted against IL-1β limits the progression of atherosclerosis in ApoE deficient mice [61]. Similar results have been reported in preclinical studies involving IL-18 [62,63]. 

Unlike other markers of inflammation, hsCRP is a validated downstream marker of inflammation that remains stable over time, has no diurnal variation and captures much of the upstream inflammatory cascade, including the aforementioned IL-1/IL-6 signaling pathway [64,65]. While initially described as a component of the acute inflammatory response in 1930, it gained traction among cardiovascular researchers in the 1990s as studies came to light showing that elevated levels of hsCRP were associated with acute coronary events, sudden death from cardiac cause and stroke [66,67,68,69]. Analysis of data from the Physician’s Health Study showed that elevated levels of hsCRP were prevalent among apparently healthy individuals prior to their first cardiovascular event [70]. Furthermore, this study also highlighted the value of assessing inflammatory state in at-risk patients, with multivariate-adjusted analyses suggesting that hsCRP was a stronger predictor of a first cardiovascular event than LDL-C level. The relationship between hsCRP and cardiovascular outcomes was later supported by a meta-analysis, which involved over 160,000 individuals from 54 long-term prospective studies amassing nearly 28,000 adverse clinical outcomes [71]. This showed that each standard deviation increase in log-normalised hsCRP was associated with an increase in relative risk of 1.37 (95% CI 1.27–1.48) for CAD and 1.55 (95% CI 1.37–1.76) for cardiovascular mortality when adjusted for traditional risk factors [71]. 

**Figure 2:**
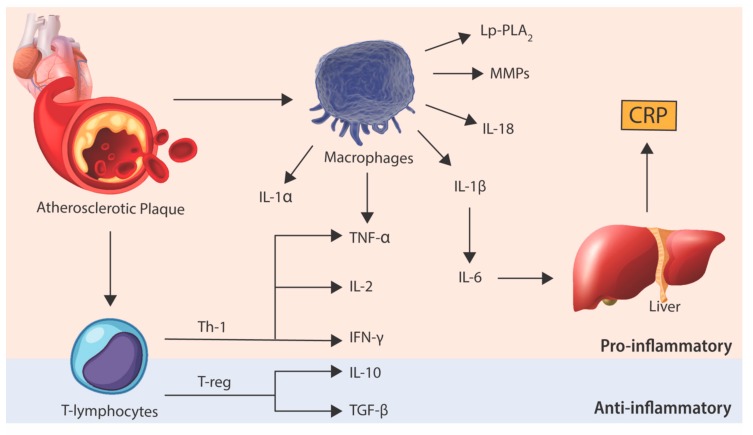
**Inflammatory pathways involved in atherosclerosis.** Data from preclinical and clinical trials suggest an intricate balance between pro-inflammatory and anti-inflammatory pathways. It is this balance that determines the development and progression of atherosclerotic plaque that may result in the thrombotic complications associated with plaque rupture. CRP, C-reactive protein; MMPs, matrix metalloproteinases; IFN-γ, interferon-gamma; IL-1α, interleukin-1-alpha; IL-1β, interleukin-1-beta; IL-2, interleukin-2; IL-6, interleukin-6; IL-10, interleukin-10; IL-18, interleukin-18; Lp-PLA_2_, lipoprotein-associated phospholipase A2; TGF-β, transforming growth factor beta; Th-1, T-helper-1 lymphocyte; TNF-α, tumor necrosis factor alpha; T-reg, regulatory T lymphocyte.

Taken into context, attempts to reduce inflammatory burden were naturally seen as the next step in further reducing cardiovascular risk in the “statin era”. Given the growing body of epidemiological and experimental data over the last two decades implicating inflammatory pathways in atherosclerosis (Figure 2), studies have now progressed into clinical trials investigating the efficacy of various novel anti-inflammatory agents in preventing atherosclerotic complications. However, the substantial costs associated with developing novel pathway specific anti-inflammatory agents represents a major barrier in translating the preclinical research into a commercial atherosclerosis treatment. Therefore, the field has also seen efforts to repurpose established agents that have broad, more pleiotropic anti-inflammatory effects to provide a cost-effective solution to address residual inflammatory risk.

## 6. A New Era: Human Trials Targeting Cytokine Inhibition

Early clinical trials of cytokine-targeted therapies for CVD were conducted in the setting of congestive heart failure, with most large studies failing to demonstrate benefit in mortality or hospitalisation rates in these patients. These studies primarily involved inhibition of TNF-α with etanercept [72], a decoy receptor that lowers circulating levels of TNF-α, and infliximab [73], a monoclonal antibody to TNF-α. Given the lack of benefit in patients with heart failure, as well as the potential for TNF-α inhibitors to adversely affect lipid profiles, interest in targeting TNF-α in atherosclerotic CVD has been limited [72,73,74,75]. To date, the most successful trials involving anti-cytokine therapy for atherosclerotic CVD have targeted IL-1β, the major circulating form of IL-1, with subsequent downstream effects on IL-6 (Table 1). The most prominent of these is the CANTOS (Canakinumab Anti-inflammatory Thrombosis Outcome Study) trial published in late 2017 [76].

### 6.1. Canakinumab: The CANTOS Trial 

CANTOS was a randomised, double blind, placebo-controlled trial of 10,061 patients with stable CAD, who had suffered a previous MI and had an elevated inflammatory state, defined as hsCRP of greater than 2 mg/L [76]. To address questions regarding residual inflammatory risk despite standard care, over 90% of study participants were on statin therapy, 80% were treated for hypertension and 40% were on treatment for diabetes mellitus. Patients were assigned to four groups that received three monthly subcutaneous injections of canakinumab, a human monoclonal antibody targeting IL-1β, at three different doses (50 mg, 150 mg or 300 mg) or placebo. Canakinumab is currently approved for rare autoinflammatory syndromes, such as Muckle–Wells syndrome, and as a second line biologic agent for rheumatoid diseases. The doses of canakinumab selected for this trial were based on a pilot study involving 566 high risk patients who had well-controlled diabetes mellitus and received doses ranging from 5 mg to 150 mg [81]. Although no change in LDL-C levels were observed in this smaller study, canakinumab reduced hsCRP levels by approximately 65% with no clear dose response effect [81]. Therefore, the higher and lower doses anchored around 150 mg used in CANTOS were selected to address questions relating to safety and dose-response. After 48 months, CANTOS participants who received 150 mg or 300 mg of canakinumab had 35% to 40% reductions in hsCRP compared to placebo, without significant reductions in LDL-C. A similar magnitude of reduction was observed in IL-6 levels measured at 12 months. 

The primary endpoint of the study was the first occurrence of a MACE, which was defined as a composite of MI, non-fatal stroke, or cardiovascular death. Canakinumab significantly reduced this endpoint to 3.86 events per 100 person-years in the 150 mg group compared to 4.5 per 100 person-years in the placebo group, translating to a 15% relative risk reduction for MACE (hazard ratio (HR) 0.85, 95% CI 0.74–0.98; *p* = 0.0208; threshold *p*-value of 0.0212). While a similar risk reduction was also observed in the 300 mg dose group, the threshold p-value for significance was not met (HR 0.86; 95% CI 0.75–0.99; *p* = 0.0314; threshold *p*-value of 0.0106). No significant benefit when compared to placebo was achieved in participants receiving 50 mg (HR 0.93; 95% CI 0.80–1.07; *p* = 0.30; threshold *p*-value = 0.0212), suggesting that doses of at least 150 mg are required for meaningful cardiovascular protection. Subgroup analysis of CANTOS participants showed that those who achieved hsCRP levels < 2 mg/L on canakinumab, termed “cytokine responders”, had a 25% reduction in MACE (HR 0.75; 95% CI 0.66–0.85; *p* < 0.0001), whereas cytokine non-responders had no significant benefit (HR 0.90; 95% CI 0.79–1.02; *p* = 0.11) [82]. Similarly, of *n* = 4833 participants who had IL-6 levels measured at baseline, those who achieved on-treatment levels below the study median of 1.65 ng/L had a striking 32% reduction in MACE (HR 0.68; 95% CI 0.56–0.82; *p* < 0.0001) [83]. Taken together, CANTOS provides robust evidence that inhibition of pro-inflammatory pathways, at least in the IL-1β/IL-6 signally cascade, can yield significant reduction in cardiovascular risk that is independent of any lipid lowering effect. Crucially, this benefit applies to individuals who have a clear inflammatory biomarker response to canakinumab and is incremental to the use of conventional CAD pharmacotherapies. 

An important consideration for all trials involving novel pharmacological therapies is adverse events. In regards to anti-inflammatory agents, the major adverse effect anticipated is reduced host immunity with subsequent increased rates of infection. In CANTOS, patients receiving canakinumab had a significantly higher incidence of fatal infection and sepsis compared to placebo (incidence rate 0.31 vs. 0.18 events per 100 person-years; *p* = 0.02). The majority of these infections were due to gram positive organisms rather than opportunistic infections or reactivation of tuberculosis. While these infections can be treated with antibiotics, the results of CANTOS highlight the importance of vigilant clinical surveillance following the administration of any anti-inflammatory agent. Furthermore, another concern of anti-inflammatory agents has the potential for cancer given the role the immune system has in cancer surveillance. In CANTOS, no significant difference in cancer incidence was observed between patients given canakinumab compared to those given placebo (*p* = 0.38). Instead, patients given canakinumab had a significantly lower cancer mortality rate than the placebo group. This benefit was predominantly due to a reduction in lung cancer rate, specifically non-small cell lung cancer [84].

Given the positive results of CANTOS with canakinumab, attention has now turned to how to best reduce residual inflammatory risk in patients with atherosclerotic CAD in real-world practice. One challenge facing the use of canakinumab is that its high cost makes it unlikely to be adopted for widespread use given the high prevalence of atherosclerotic CAD [2]. Given the multifarious nature of inflammation in atherosclerotic processes, an attractive alternative strategy is the repurposing of established, generic drugs that have broad anti-inflammatory properties for atheroprotection. Two potential candidates that have been investigated in clinical trials are methotrexate and colchicine. 

### 6.2. Methotrexate: The Cardiovascular Inflammation Reduction Trial (CIRT) Trial

In contrast to CANTOS which employed a relatively narrow-spectrum, pathway-specific approach to targeting inflammation, CIRT (Cardiovascular Inflammation Reduction Trial) was aimed at determining whether low dose methotrexate, a broad-spectrum anti-inflammatory drug, could also achieve reductions in cardiovascular events [77]. Methotrexate is an established, inexpensive drug with pleiotropic upstream anti-inflammatory activity that is used as first line therapy for conditions characterised by systemic inflammation, such as rheumatoid arthritis and psoriasis. In contrast to high dose methotrexate which acts via an anti-proliferative cytotoxic mechanism, low dose methotrexate regulates inflammation through the inhibition of aminoimidazole-4-carboximide ribonucleotide (AICAR), which results in increased levels of adenosine. This in turn is thought to down-regulate inflammation through several mechanisms including the suppression of proinflammatory cytokines such as IL-12, IL-6 and TNF-α, upregulating the production of anti-inflammatory cytokines such as IL-10 and IL-1 receptor antagonist, and down-regulating macrophage activation and the T-helper-1 response [85,86]. The CIRT trial was designed on a background of several observational studies showing that methotrexate used for rheumatoid conditions was associated with lower CVD risk [87,88,89], including a meta-analysis [90] that had found a 21% lower risk for total CVD and 18% lower risk of MI specifically.

CIRT randomised 4786 participants with a history of previous MI and either type 2 diabetes or metabolic syndrome to methotrexate at a target oral weekly dose of 15 to 20 mg or placebo. Similar to CANTOS, both study groups also received standard care, with over 85% of participants on statin therapy. After a median follow-up of 2.3 years, low dose methotrexate did not significantly reduce IL-1β, IL-6 or hsCRP levels compared to placebo. Furthermore it did not significantly impact the primary endpoint of the first occurrence of MACE, defined as a composite of non-fatal MI, non-fatal stroke and cardiovascular death (HR 1.01; 95% CI 0.82–1.25; *p* = 0.91), nor the secondary composite endpoint that comprised MACE and unstable angina presentations requiring unplanned revascularisation (HR 0.96; 95% CI 0.79–1.15; *p* = 0.67). 

In trying to account for the discrepant results between CIRT and CANTOS, two key differences between these studies warrant specific mention. Firstly, an elevated hsCRP level was not an inclusion criterion in CIRT participants in contrast to CANTOS. The median baseline hsCRP was 1.5 mg/L in CIRT compared to 4.2 mg/L in CANTOS. This difference provides an important insight suggesting that attempts to mitigate cardiovascular risk with anti-inflammatory therapies may only be effective in patients with atherosclerosis who have an elevated inflammatory signal and therefore residual inflammatory risk, despite standard care. Secondly, CANTOS which used canakinumab to specifically target IL-1β reported a significant decrease in IL-1β, IL-6 and hsCRP levels. Low dose methotrexate, on the other hand, showed no effect on biomarkers of the IL-1β signaling pathway and failed to reduce hsCRP, a downstream non-specific marker of inflammatory state. This suggests that this treatment approach had minimal effect, if any, on the IL-1β/IL-6 signaling pathway. The neutral results from CIRT are consistent with previous clinical trials that also failed to show benefit from other anti-inflammatory approaches to treating atherosclerosis. These include studies of losmapimod [80], a p38 MAP (mitogen-activated protein) kinase inhibitor, and darapladib [79], an inhibitor of lipoprotein-associated phospholipase A2 (Lp-PLA_2_). Elevated levels of Lp-PLA_2_ and p38 MAP kinase-mediated immune activation have been hypothesised to be involved in the pathogenesis of atherosclerosis [91]. Therefore, not all therapies with putative anti-inflammatory effects are capable of achieving clinically significant outcomes in patients with CAD, with the CANTOS results highlighting that the IL-1β/IL-6 pathway is an especially promising target.

### 6.3. Alternative Targets in the IL-1β/IL-6 Signaling Pathway

**Figure 3:**
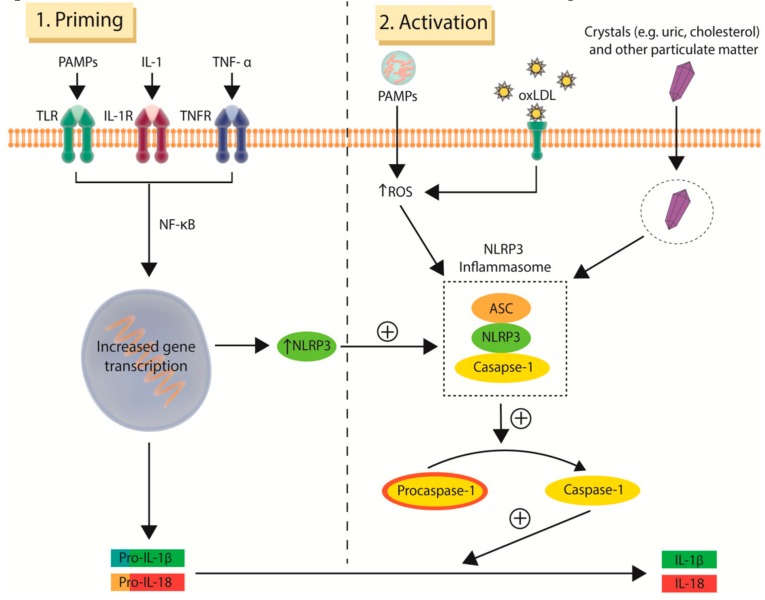
**Two signal activation of the NOD-like receptor family pyrin domain containing 3 (NLRP3) inflammasome.** Activation of the NLRP3 inflammasome requires both the initial priming step (left) following by the activation step (right). Priming of macrophages is provided by endogenous cytokines (IL-1 and TNF-α) and binding of PAMPs on microbial molecules to membrane-bound TLR. The second activation step involves a variety of stimuli, including intracellular oxLDL accumulation, PAMPs, and crystalloid particulates such as uric and cholesterol crystals. The NLRP3 inflammasome catalyses the conversion of pro-caspase-1 to its active form, caspase-1, which then converts pro-IL-1β and pro-IL-18 into their bioactive, pro-atherogenic forms, IL-1β and IL-18. ASC, apoptosis-associated speck-like protein; IL-1, interleukin 1; IL-1R, interleukin-1 receptor; NLRP3, NOD-like receptor family pyrin domain containing 3; oxLDL, oxidised low-density lipoproteins; PAMPs, pathogen-associated molecular patterns; ROS, reactive oxygen species; TLR, toll-like receptors; TNF-α, tumor necrosis factor alpha; TNFR, tumor necrosis factor receptors.

The encouraging results of CANTOS, which identified IL-1β as an effective target for mitigating residual inflammatory risk, represent a major stimulant to an already nascent field. It is therefore not surprising that attention has moved up the cytokine stream to target inflammasomes of which NLRP3, a known activator of IL-1β, is best characterised [92]. The various mechanisms leading to inflammasome activation and assembly are still an area of ongoing research with numerous pathways identified to date (Figure 3), one of which has been shown to occur following the exposure of neutrophils and macrophages to cholesterol crystals in atherosclerotic plaque [93,94]. OxLDL and other types of crystals, including uric acid crystals as seen in gout, are also known to activate the pro-inflammatory NLRP3 response [93,95]. Alone, these activators are unable to initiate the assembly of the NLRP3 inflammasome unless an initial priming step occurs [96]. This is thought to be due to low protein levels of NLRP3 in unprimed macrophages. Priming of macrophages is mediated by NF-κB signaling with the stimulus provided by endogenous cytokines, such as IL-1 and TNF-α, as well as pathogen-associated molecular patterns (PAMPs) on microbial molecules that interact with toll-like receptors (TLR). This in turn upregulates the expression of NLRP3 and pro-IL-1β thus providing the reactants required for NLRP3 inflammasome formation and function (Figure 3) [97]. 

To date, animal studies utilising NLRP3 inhibitors in cardiovascular disease models have shown encouraging results [98,99]. One such study showed significant reductions in the development of atherosclerotic lesion size as well as VCAM-1 and ICAM-1 mRNA expression in hyperlipidaemic mice when given MCC950, a selective NLRP3 inhibitor, compared to controls [99]. While macrophage infiltration of plaque was found to be reduced, possibly due to reduced monocyte adhesion via VCAM-1 and ICAM-1 downregulation, further research is required to fully elicit the mechanisms underlying these findings. Reduction in infarct size and preservation of left ventricular ejection fraction have also been reported for MCC950 in a pig model of MI [98]. 

Inhibition of IL-6, which is downstream of IL-1β, is also seen as a potential target for anti-inflammatory intervention. Early experimental data implicating IL-6 in atherosclerosis demonstrated that exogenous injection of IL-6 into hyperlipidaemic mice significantly increased atherosclerotic lesion size [100]. Subsequently, recent data have shown that administration of MR16-1, a murine IL-6 receptor antibody, yields significant atheroprotection in hyperlipidaemic mice [101]. Therefore, agents that target IL-6 binding, such as tocilizumab, or alter IL-6 receptor activity, such as sarilumab, warrant investigation for atheroprotective benefits in patients with atherosclerosis. Promising results from a small randomised trial showed that a single dose of tocilizumab reduced troponin levels in patients presenting with acute coronary syndrome, suggesting a possible reduction in infarct size [102]. However, while tocilizumab is currently used clinically in patients with rheumatoid arthritis, it has been associated with increasing LDL-C and weight gain [103,104], which in theory could promote atherosclerosis and potentially negate any long-term benefit gained from its anti-inflammatory actions. 

### 6.4. Colchicine: The LoDoCo Trial 

Another emerging candidate for repurposing in atherosclerosis is colchicine, an anti-inflammatory drug used to treat gout and other inflammatory disorders, such as familial Mediterranean fever (FMF) and recurrent pericarditis. Colchicine binds to microtubule ends and inhibits cytoskeletal microtubule processes [105]. Therefore, it affects several microtubule-dependent processes such as neutrophil chemotaxis, phagocytosis and protein excretion. It has also been shown to limit the expression of adhesion molecules on the surface of leukocytes and endothelial cells [106]. Furthermore, a recent advance has been the discovery of attenuated activation of the NLRP3 inflammasome in colchicine-treated neutrophils and macrophages in response to monosodium urate crystals in the setting of gout [95,105]. This has led to speculation that colchicine can also mitigate cholesterol crystal-induced inflammation via NLRP3 attenuation within atherosclerotic plaque. 

Since Food and Drug Administration (FDA) approval in 2009 for use in gout and FMF, numerous studies have reported that colchicine may be beneficial in different cardiovascular disease states [107]. This is consistent with retrospective observations that found a lower rate of MI in patients with gout and FMF who are treated with colchicine [108,109]. Building upon this growing body of evidence, the LoDoCo (Low Dose Colchicine for Secondary Prevention of Cardiovascular Disease) study was a placebo-controlled, observer-blinded trial that randomised 532 patients with stable atherosclerosis to receive either 0.5 mg/day of colchicine or placebo in addition to standard care [78]. After a median follow-up of 3 years, participants who were given colchicine had a marked reduction in a composite endpoint consisting of MI, cardiac arrest or non-cardioembolic stroke compared to placebo (HR 0.33; 95% CI 0.18–0.59; *p* < 0.001). Although the mechanism of benefit of colchicine was not investigated in this trial, colchicine has been shown to acutely lower the local production of NLRP3 inflammasome-related cytokines, IL-1β, IL-18 and IL-6, in the coronary vascular bed of patients with acute coronary syndrome [110]. Furthermore, a recent study using coronary computed tomography (CT) angiography found that regular colchicine use at a dose of 0.5 mg/day favourably modified the composition of coronary atherosclerotic lesions by reducing low attenuation plaque volume, a known predictor of future MACE [111]. Finally, similar to canakinumab and in contrast to methotrexate, colchicine has been shown to lower hsCRP by more than 50% in patients who have stable CAD with elevated levels of inflammation (hsCRP ≥ 2 mg/L), incrementally to statin therapy [112]. These results have sparked five registered clinical trials (Table 2) of colchicine that are currently recruiting patients with stable CAD (LoDoCo II), unstable CAD (Colchicine Cardiovascular Outcomes Trial (COLCOT), Colchicine for Acute Coronary Syndromes (COACS), Colchicine and Spironolactone in Patients with STEMI/SYNERGY Stent Registry (CLEAR-SYNERGY)) and cerebrovascular disease (CONVINCE). 

Repurposing of colchicine for use in atherosclerosis is not without its own challenges given its well-known propensity to cause gastrointestinal side-effects in up to 25% of patients [113,114]. Therefore, definitive proof of efficacy, as well as further mechanistic understanding of how colchicine attenuates cardiovascular risk in CAD will facilitate physician confidence in prescribing this drug, as was the case with statins during their early development. 

## 7. Clonal Haematopoiesis: A Novel Risk Factor for Atherosclerosis 

Clonal haematopoiesis of indeterminant potential (CHIP) occurs with normal ageing and is the result of accumulated somatic mutations in bone marrow stem cells [115,116]. These somatic mutations provide selective advantages over unaffected stem cells resulting in an increased relative proportion of these clones in the peripheral circulation without causing any other haematological abnormalities. The most common genes affected by mutations in CHIP, in order of decreasing frequency, are the epigenetic regulars DNMT3A (DNA methyltransferase 3 alpha), TET2 (Tet methylcytosine dioxygenase 2) and ASXL1 (additional sex combs like 1). Results from cohort studies confirm that CHIP is an age-related disorder affecting up to 16.4% of people aged over 80, compared to less than 1% of those under the age of 40 [115]. While CHIP conveys an increased risk of haematological malignancy (HR 11.1; 95% CI 3.9–32.6), carriers of its associated mutations are also 1.9 times more likely than non-carriers to have CAD (95% CI 1.4–2.7) and 4 times more likely to have an MI (95% CI 2.4–6.7) [117]. Therefore, CVD may account for a disproportionate number of non-haematological mortalities in CHIP carriers. 

Attempts to investigate CHIP in preclinical studies have shown promising results that link its associated mutations to the development of atherosclerosis. Atherosclerosis-prone mice transplanted with TET2-deficient haematopoietic stem cells developed larger atherosclerotic lesions than controls without significant differences in lipid levels [117]. Analysis of macrophages extracted from these TET2-deficient mice showed increased levels of pro-inflammatory cytokines, including IL-1β and IL-6. Furthermore, in a murine model of heart failure, TET2 deficiency-associated cardiac dysfunction was associated with a corresponding increase in IL-1β levels. Interestingly, in this study treatment with MCC950, a selective NLRP3 inhibitor, protected against the development of heart failure in TET2-deficient mice [118]. Therefore, further research on CHIP and its associated mutations may provide yet another target for future therapeutic agents to attenuate the associated increased cardiovascular risk. 

## 8. Conclusions

Positive lifestyle interventions, such as weight control, smoking cessation and physical exercise, remain the basis of primary prevention of CVD and should accompany any pharmacological therapy aimed at addressing cardiovascular risk. Despite this, cardiovascular events continue to occur at alarming rates with residual inflammation recently identified as a treatable pathogenic factor. Results from the CANTOS trial establish that inflammation, specifically via the IL-1β/IL-6 signally pathway, is a therapeutic target capable of achieving clinically significant benefits in those shown to have residual inflammation despite contemporary standard care. With numerous clinical trials investigating other anti-inflammatory agents underway, it is clear that CANTOS represents only the beginning of what promises to be an exciting and rapidly moving era of research in atherosclerotic CVD. The recent discovery of CHIP as another novel risk factor for CVD further highlights the existence of atherogenic pathways that are independent of traditional risk factors. With inflammation now clearly shown to be an important mediator and risk factor in atherosclerosis, it is these previously unsuspected pathways that may serve as the future frontier for cardiovascular risk modification. 

## Figures and Tables

**Table 1:** Major published clinical trials involving anti-inflammatory agents in atherosclerotic heart disease.

Trial Name	Study Design	Patient Number	Intervention	Primary Outcomes	Results	Benefit Observed
**Broad-spectrum anti-inflammatory approach**						
**Methotrexate**						
Cardiovascular Inflammation Reduction Trial (CIRT) [77]	Phase 3 multicentre, randomised, double-blind, placebo-controlled	4786	Oral low methotrexate (target dose of 15–20 mg weekly) vs. placebo	Non-fatal myocardial infarction, non-fatal stroke and cardiovascular death	HR 1.01; 95% CI 0.82–1.25; *p* = 0.91	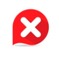
**Colchicine**						
Low-dose colchicine for secondary prevention of cardiovascular disease (LoDoCo) [78]	Phase 3 multicentre, randomised, double-blind, placebo-controlled	532	Colchicine 0.5 mg/day vs. placebo	MI, fatal or non-fatal out-of-hospital cardiac arrest, or non-cardioembolic ischaemic stroke	HR 0.33; 95% CI 0.18–0.59; *p* < 0.001	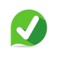
**Narrow-spectrum anti-inflammatory approach**						
**IL-1β**						
Anti-inflammatory Therapy with Cankinumab for Atherosclerosis (CANTOS) [76]	Phase 3 multicentre, randomised, double-blind, placebo-controlled	10,061	Subcutaneous injection of canakinumab (50 mg, 150 mg or 300 mg) every 3 months vs. placebo	Non-fatal MI, non-fatal stroke and cardiovascular death	HR 0.85; 95% CI 0.74–0.98; *p* = 0.021 in the 150 mg-treated group	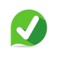
**Lipoprotein-associated phospholipase A2 (Lp-PLA_2_)**						
SOLID-TIMI 52 [79]	Phase 3 multicentre, randomised, double-blind, placebo-controlled	13,026	Daily oral darapladib 160 mg vs. placebo	Coronary heart disease death, non-fatal MI and urgent revascularisation for myocardial ischaemia	HR 1.00; 95% CI 0.91–1.09; *p* = 0.93	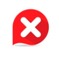
**P38 mitogen-activated protein kinase (MAPK)**						
LATITUDE-TIMI 60 [80]	Phase 3 multicentre, randomised, double-blind, placebo-controlled	3503	Oral losmapimod 7.5 mg twice daily vs. placebo	Non-fatal MI, severe recurrent ischaemia requiring urgent coronary artery revascularisation and cardiovascular death	HR 1.16; 95% CI 0.91–1.47; *p* = 0.24	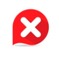

Abbreviations: CI, confidence interval; HR, hazard ratio; IL, interleukin; mg, milligrams; MI, myocardial infarction. The green tick sign represents that the intervention was associated with a significant benefit whereas a red cross represents that there was no significant benefit observed.

**Table 2:** Ongoing clinical studies involving colchicine in cardiovascular disease.

Trial Name	Primary Site(s)	Study Design	Patient Number	Intervention	Primary Outcomes	Follow-up	Completion Date
**Stable coronary artery disease**							
LoDoCo II: Low-dose Colchicine for Secondary Prevention of Cardiovascular Disease (ACTRN12614000093684)	Australia, Netherlands	Phase 3 multicentre, double blind, randomised placebo-controlled	5500	Colchicine 0.5 mg/day vs. placebo	ACS, cardiovascular death or stroke	3 years	20 Jan
**Acute Coronary Syndrome (ACS)**							
COLCOT: Colchicine Cardiovascular Outcomes Trial (NCT02551094)	Canada	Phase 3 randomised placebo-controlled	4745	Colchicine 0.5 mg/day vs. placebo	MI, cardiovascular death, resuscitated cardiac arrest, stroke, or angina pectoris requiring revascularisation	3–4 years	19 Sep
COACS: Colchicine for Acute Coronary Syndromes (NCT01906749)	Italy	Phase 4 multicentre, double blind, randomised placebo- controlled	500	Colchicine 0.5 mg/day vs. placebo	ACS, ischaemic stroke, and overall mortality	2 years	N/A
CLEAR-SYNERGY (OASIS-9): Colchicine and Spironolactone in Patients with STEMI/SYNERGY Stent Registry (NCT03048825)	Canada	Phase 3 multicentre, blinded, randomised placebo-controlled. 4 study arms, 2 × 2 factorial design	4000	Colchicine 1 mg/day and/or spironolactone 25 mg/day and/or placebo and/or SYNERGY stent	Cardiovascular death, recurrent MI, or stroke in the colchicine-treated group	2 years	21 Dec
**Cerebrovascular disease**							
CONVINCE: Colchicine for Prevention of Vascular Inflammation in Non-cardio Embolic Stroke (NCT02898610)	Belgium, Ireland, Greece and Spain	Phase 3 multicentre, open-label, placebo controlled	2623	Colchicine 0.5 mg/day vs. placebo	Non-fatal major cardiac event and vascular death	5 years	21 Oct

Abbreviations: ACS, acute coronary syndrome; MI, myocardial infarction.
